# Dual-Phase Cardiac Diffusion Tensor Imaging with Strain Correction

**DOI:** 10.1371/journal.pone.0107159

**Published:** 2014-09-05

**Authors:** Christian T. Stoeck, Aleksandra Kalinowska, Constantin von Deuster, Jack Harmer, Rachel W. Chan, Markus Niemann, Robert Manka, David Atkinson, David E. Sosnovik, Choukri Mekkaoui, Sebastian Kozerke

**Affiliations:** 1 Institute for Biomedical Engineering, University and ETH Zurich, Zurich, Switzerland; 2 Department of Mechanical and Biomedical Engineering, Massachusetts Institute of Technology, Cambridge, Massachusetts, United States of America; 3 Imaging Sciences and Biomedical Engineering, King's College London, London, United Kingdom; 4 Centre for Medical Imaging, University College London, London, United Kingdom; 5 Department of Cardiology, University Hospital Zurich, Zurich, Switzerland; 6 Department of Radiology, University Hospital Zurich, Zurich, Switzerland; 7 Athinoula A. Martinos Center for Biomedical Imaging, Department of Radiology, Massachusetts General Hospital, Harvard Medical School, Boston, Massachusetts, United States of America; 8 Cardiovascular Research Center, Cardiology Division, Massachusetts General Hospital, Harvard Medical School, Boston, Massachusetts, United States of America; 9 Department of Radiology, University Hospital Center of Nîmes, EA 2415, Nîmes, France; 10 Faculty of Medicine, Montpellier 1 University, Montpellier, France; University Medical Center Utrecht, Netherlands

## Abstract

**Purpose:**

In this work we present a dual-phase diffusion tensor imaging (DTI) technique that incorporates a correction scheme for the cardiac material strain, based on 3D myocardial tagging.

**Methods:**

*In vivo* dual-phase cardiac DTI with a stimulated echo approach and 3D tagging was performed in 10 healthy volunteers. The time course of material strain was estimated from the tagging data and used to correct for strain effects in the diffusion weighted acquisition. Mean diffusivity, fractional anisotropy, helix, transverse and sheet angles were calculated and compared between systole and diastole, with and without strain correction. Data acquired at the systolic sweet spot, where the effects of strain are eliminated, served as a reference.

**Results:**

The impact of strain correction on helix angle was small. However, large differences were observed in the transverse and sheet angle values, with and without strain correction. The standard deviation of systolic transverse angles was significantly reduced from 35.9±3.9° to 27.8°±3.5° (p<0.001) upon strain-correction indicating more coherent fiber tracks after correction. Myocyte aggregate structure was aligned more longitudinally in systole compared to diastole as reflected by an increased transmural range of helix angles (71.8°±3.9° systole vs. 55.6°±5.6°, p<0.001 diastole). While diastolic sheet angle histograms had dominant counts at high sheet angle values, systolic histograms showed lower sheet angle values indicating a reorientation of myocyte sheets during contraction.

**Conclusion:**

An approach for dual-phase cardiac DTI with correction for material strain has been successfully implemented. This technique allows assessing dynamic changes in myofiber architecture between systole and diastole, and emphasizes the need for strain correction when sheet architecture in the heart is imaged with a stimulated echo approach.

## Introduction

The influence of myocardial fiber architecture on cardiac morphology and mechanics is of significant interest. The helical organization of myocardial fibers [1]–[4] and the formation of myocytes into sheets [5]–[7] by branching and interconnection have been well described. While microscopy provides high-resolution images [8]–[10], MR diffusion weighted imaging enables investigation of the intact organ. Despite its lower spatial resolution compared to microscopy, MR has found application in *ex vivo* [4], [6], [11]–[20] and also in a small number of *in vivo* [21]–[27] studies of the heart. More recently, diffusion tensor imaging (DTI) has enabled tractography of the myocardium both *ex vivo* [28]–[30] and *in vivo* [25], [31], [32].

In order to investigate differences in fiber configuration during the cardiac cycle, Chen et al. [33] presented a comparison of *ex vivo* pig hearts first arrested in diastole and later fixated in systole. These *ex vivo* findings were confirmed by Hales et al. [34]. Significant differences in helix angle and sheet angle distributions between systole and diastole were found. Further investigations involved the study of sheet rearrangement in myocardial pathologies [35], [36].

*In vivo* DTI of the human and animal heart has been performed using diffusion weighted stimulated echo acquisition modes (STEAM) [21], [27], [37]–[40] and first and second order motion compensated diffusion weighted spin-echoes [24]–[26], [31], [32] in combination with echo-planar imaging readouts. While spin-echo diffusion weighted imaging requires strong gradient systems in order to be applied in the *in vivo* heart, STEAM based sequence can be performed with standard gradient hardware.

Early reports suggested that the impact of material strain is of significance in cardiac DTI [21], [22], [38], [39]. To this end, it was proposed to trigger STEAM encoding and decoding to the so-called “sweet spots” of myocardial strain at which the temporal mean of strain approaches zero [21]. Despite this insight, cardiac diffusion-weighted STEAM at various time points in the cardiac cycle has been reported recently [27], [41]–[44].

The objective of the present work is to address the impact of material strain on the diffusion tensor when imaging the in-vivo heart using the STEAM sequence. To this end, a tensor correction scheme based on cine 3D tagging data is presented. In addition, the diffusion weighted STEAM sequence is modified to allow for dual-phase and slice-interleaved imaging thereby accelerating cardiac diffusion tensor imaging by a factor of two relative to previous single-phase approaches. Differences in fiber and sheet architecture between systole and diastole, without and with strain correction of the in-vivo human heart are presented.

## Methods

### Study protocol

Ten subjects without any history of cardiac disease (4 male/6 female, age 27±8years, weight 68±7 kg, heart rate 66±11 bpm) were imaged on a clinical 1.5T scanner (Achieva system, Philips Healthcare, Best, The Netherlands). The scanner was equipped with a gradient system delivering 40 mT/m maximum strength and 200 mT/m/ms maximum slew rate per physical gradient axis. A 5-channel cardiac array coil was used for signal detection.

Written informed consent was obtained from each subject prior to imaging, and the study protocol was approved by the ethics committee of the canton of Zurich. Obtained informed consent included imaging as well as publication of anonymized data.

Short-axis balanced steady state free precession cine data with a temporal resolution of 7 ms were obtained in the 2-chamber and short-axis planes of the left ventricle (LV) to identify the ventricular systolic (trigger delay: 277±19 ms) and diastolic (trigger delay: 627±85 ms) standstill periods. For image-based shimming, a B0 field map was acquired in the short-axis view covering the entire LV [45].

### Myocardial tagging sequence

Three orthogonally orientated line tagged cine image volumes covering the entire LV were acquired within three consecutive breath holds [46]. To compensate for differences in breath hold levels a gating respiratory navigator was applied prior to the acquisition of each stack (acceptance window 15 mm). Resulting navigator offsets were used for stack alignment during image reconstruction. In order to avoid tag line fading during the cardiac cycle, complementary spatial modulation of magnetization (CSPAMM) was applied requiring two signal averages, with inverted tagging modulation [47]. Imaging parameters were as follows: FOV: 108×108×108 mm^3^, spatial resolution: 3.5×7.7×7.7 mm^3^ (tagging/readout × phase encoding × phase encoding) reconstructed to 0.96×0.96×0.96 mm^3^, temporal resolution: 18 ms and 7 mm tag line distance. To achieve a temporal resolution of 18 ms, the maximum slew rate of the gradient system (200 mT/m/ms) was used for the segmented echo planar imaging-readout (EPI factor 7, 3 excitations per heart phase).

### Diffusion sequence

**Figure 1:**
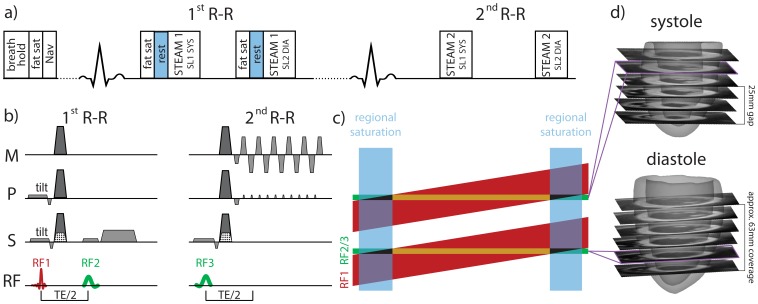
Dual-phase cardiac DTI acquisition scheme. The first slice is encoded in systole (STEAM 1 SL1 HP1) and the second slice in diastole (STEAM 1 SL2 HP2) with preceding fat saturation (fat sat) and regional saturation (rest) (a). Corresponding STEAM decoding and readout are performed in the second R-R interval (STEAM 2). For non-diffusion weighted imaging, FID crushers are applied in the through-plane direction (dotted area) (b) while only diffusion encoding gradients are applied otherwise (dark gray). (C) Non-coplanar excitation (tilt) is used to select two angulated slabs (red) with the first RF pulses. Slice selection within these slabs is performed with the second and third RF pulses (green). Regional saturation (blue) is used to eliminate signal from the edges (black). The final slices are represented in brown, and the measured slice distribution across the left ventricle is shown in (d). The coverage from apex to base was approximately 63 mm.

Dual-phase cardiac STEAM was implemented using a reduced field-of-view technique [48]. To avoid saturation in adjacent slices a tilted local-look pulse scheme was incorporated (Figure 1) [49]. Residual signal from the edge of the field-of-view (black triangles in Figure 1c) was suppressed using regional saturation (rest) slabs. A scheme to interleave slices (SL1 and SL2) and heart phases (SYS and DIA) was implemented by applying the STEAM encoding block of slice 1 in systole (SL1 SYS) and slice 2 in diastole (SL2 DIA) of the first R-R interval. The corresponding STEAM decoding block including the readout was applied in the second R-R interval, respectively. The two paired slices had a gap of 25 mm, thus avoiding cross talk from the angulated excitation due to contraction of the heart. After acquiring all signal averages of all diffusion encoding directions, the slice order was switched to complete the acquisition of both heart phases for each slice. Thereby two slices were acquired in two heart phases within a single scan, hence reducing scan time relative to sequential single-phase, single-slice acquisition by a factor of two.

The acquisition of diffusion-weighted images was divided into multiple navigator-gated breath holds (acceptance window of 5 mm). Parameters of the diffusion sequence were: 224×100 mm^2^ field-of-view, 2×2 mm^2^ in-plane resolution, 8 mm slice thickness, number of slices 6, TE/TR 18 ms/2R-R intervals, partial Fourier factor 0.62. A single shot EPI readout was used. The two heart phases in the diffusion protocol were triggered to quiescent phases of systole and mid-diastole as defined on the cine images. Unipolar diffusion gradients were played out in 10 directions on a unit-sphere [50] with a b-value of 500 s/mm^2^. To reduce echo time, FID crushers necessary in STEAM were removed for b = 500 s/mm^2^ acquisitions, but kept for the b = 0 s/mm^2^ acquisition.

All eight signal averages of a diffusion weighted image were acquired within one breath hold. Different diffusion weightings were obtained in consecutive breath holds. The volunteers were allowed sufficient time to recover in-between breath holds to ensure consistent heart rates among the data series. A total of 11 breath holds of 14–16 s duration each were acquired per slice and two heart phases, resulting in a 15–18 min total net acquisition time for six slices at two heart phases.

### Tensor reconstruction

To compensate for residual slice mismatch due to inconsistency in breath hold levels within the 5 mm gating window and to account for eddy-current induced geometrical distortions, all diffusion weighted images were registered to the b = 0 image by means of affine image registration (elastix toolbox [51]).

Systolic and diastolic diffusion tensors were estimated based on the modified Stejskal-Tanner equations. To account for non-zero diffusion weighting of the “b = 0 s/mm^2^” scan due to diffusion weighting introduced by the FID crusher gradients present in STEAM, the signal equation was modified as:
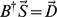
with *B* being the modified b-matrix containing the b-values of the diffusion weighted images *b* and the “b = 0” image 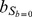:
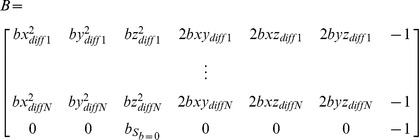


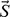
 denotes the negative logarithm of the measured signal per pixel including the signal of the “b = 0” image 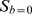:
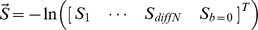
and 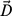 the vector containing the unknown tensor elements and the true b = 0 signal 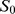:
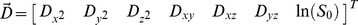
where x, y and z are the coordinates of the normalized diffusion direction, *T* the transpose and _†_ the Moore-Penrose pseudo inverse.

### Correction for material strain

Material strain effects were compensated for based on the stretch history of tissue [38], [39]. From 3D tagging data, three-dimensional displacement fields were calculated with a custom-made software utilizing the 3D SinMod algorithm [52]. The LV was manually masked on the tagging data as well as on the acquired b = 0 s/mm^2^ image. To compensate for mismatch and different spatial resolution of the acquired data, the shape of the DTI mask was mapped onto the re-sliced tagging mask by means of coherent point drift registration [53]. Having identified the position of each diffusion tensor estimated from the DTI acquisition within the displacement field, a cube parallel to the canonical basis in which the displacement fields and the position of the tensors are represented, was defined at each point and tracked over the R-R interval. The time course of the right stretch tensor was calculated from the deformation gradient field obtained from the tracked cubes as described by Hess et al. [54]. Stretch tensors were calculated relative to the systolic and the diastolic time points of diffusion imaging. The strain effect on the diffusion measurement is described by
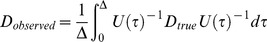
with *U*(*τ*) being the time course of the right stretch tensor and Δ the duration of the R-R interval. In accordance with [39] the equation is expanded as:
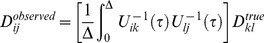
For validation purposes, additional DTI data were acquired during the systolic “sweet-spot” (trigger delay: 160 ms) in one of the volunteers and compared to data in end systole (trigger delay: 305 ms) and diastole (trigger delay: 620 ms).

### Data analysis

**Figure 2:**
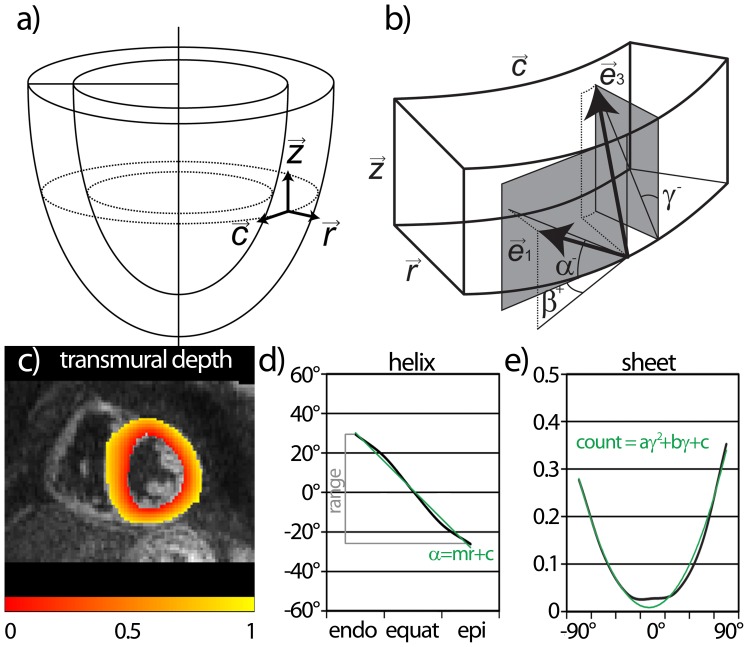
Definition of fiber and sheet angles. A local orthonormal basis is defined (a) (radial: 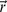, circumferential: 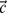, longitudinal: 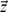). Helix (α), transverse (β) and sheet angle (γ) definitions are given in (b). For angle calculation, projections of the first (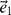) and third (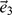) eigenvectors were used (grey planes). The sign indicates the polarity of the angle. For each tensor position a normalized transmural position is defined (c). An example of the transmural course of helix angles is shown in (d) with the angle range (grey) and linear fit (green) indicated. Histograms of sheet angles (e) were fitted using a quadratic function (green line).

From the six slices pairs of two were grouped for the basal, medial and apical level and mean diffusivity (MD) and fractional anisotropy (FA) in systole and diastole were compared for each volunteer. Additionally, helix, transverse and sheet angles were calculated using projections of the first and third eigenvectors as described in Figure 2 (a–b) [3]. To allow tracking the transmural course of the helix angle, a local anatomical basis was defined. To this end, the shape of the LV was mapped onto an “ideal” circular ring by means of coherent point drift mapping [53]. This procedure allowed the definition of a locally normalized transmural position independent of variation of local thickness of the myocardium (Figure 2c). The helix angle alignment was analyzed on a slice-by-slice basis. Therefore the myocardium was separated into five layers: epicardial, sub-epicardial, mid-wall, sub-endocardial and endocardial similarly to previously reported helix angle analysis [27]. The transmural helix gradient from the linear fit as well as the range of the transmural course of the helix angle are reported for basal, medial and apical levels (Figure 2d). Transverse and sheet angle distributions are analyzed by means of histograms for each slice similar to the analysis of Hales et al. [34]. The standard deviation of the transverse angle distribution is reported as measure of coherence. Sheet angle distributions were fitted with a quadratic function (Figure 2e) and the coefficient of the quadratic component is presented as a measure of sheet realignment during systolic contraction. Additionally the mean of the absolute value of the sheet angle was calculated for basal, medial and apical levels in systole and diastole.

The tensors shown in this study have not been interpolated as in previous works [25], [31]. Statistical differences between systolic and diastolic values were tested using a two-tailed paired student's t-test. A p-value<0.05 was considered statistically significant. All tests were Bonferroni-corrected for multiple testing.

## Results

Dual-phase cardiac DTI data was successfully acquired in all subjects. Total exam time including subject preparation was 1.5–2 hours.

**Figure 3:**
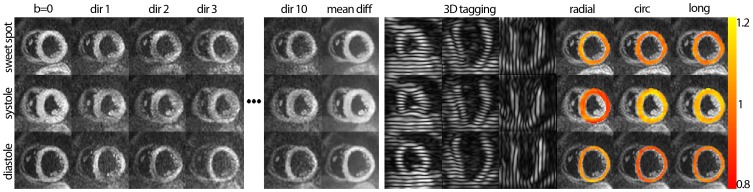
Raw data of diffusion weighted and tagging acquisitions as well as strain maps for the sweet spot, the systolic and the diastolic heart phase. The “b = 0” image, the first three and the last diffusion encoding directions as well as the averaged diffusion weighted images are shown. Tagging data from the three orthogonally oriented line-tagged stacks are given alongside. The temporally averaged stretch tensors as calculated from the tagging data allow assessing radial, circumferential and longitudinal stretch components, which are presented as stretch maps, to be assessed.

The raw data images acquired at the systolic sweet spot, in peak systole and in diastole are presented in Figure 3. Data are shown for the “b = 0”, the first three and the last diffusion- encoding direction as well as the average of all diffusion directions. Tagging data from the three orthogonally line-tagged stacks are given alongside. The temporally averaged stretch tensors as calculated from the tagging data allow radial, circumferential and longitudinal stretch components, which are presented as stretch maps, to be assessed.

**Figure 4:**
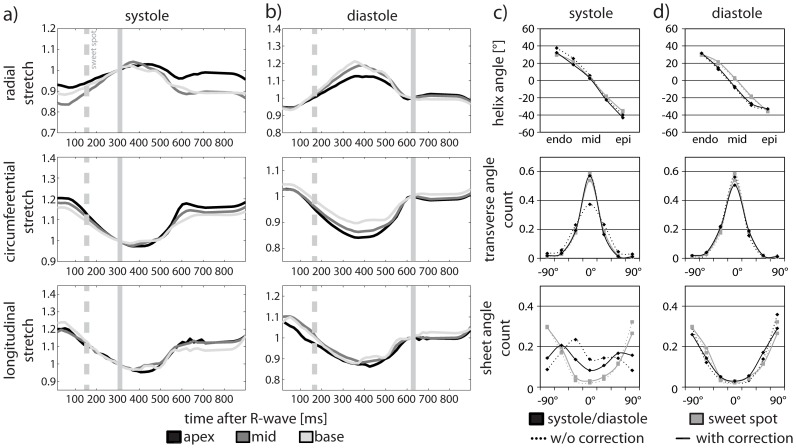
Time course of the measured stretch tensor. The radial, circumferential and longitudinal components of the right stretch tensors are plotted as a function of time after the R-wave. The systolic (a) and diastolic (b) timing of the DTI sequence is indicated by the vertical solid line while the systolic sweet spot is marked by the vertical dashed line. The transmural course of the helix angles and the transverse and sheet angle histograms are presented for systole (c) and diastole (d) for a medial/basal level. Systolic and diastolic (black) as well as sweet-spot (gray) data are shown before (dotted line) and after (solid line) strain correction.

Figure 4a,b shows the time course of the radial, circumferential and longitudinal stretch calculated from the right stretch tensors at the apical, medial and basal levels for systole and diastole. The time points of acquisition of systolic and diastolic DTI data as well as the systolic sweet spot are indicated by vertical lines in Figure 4a,b. The transmural course of the helix angles and transverse and sheet angle histograms in systole (trigger delay: 305 ms) and diastole (trigger delay: 620 ms) (without and with strain correction) are shown for the medial level in Figure 4c,d. In addition, DTI data acquired in the systolic sweet spot (trigger delay: 160 ms) is overlaid. Strain correction results in changes in the transverse and sheet angle distributions in systole. Values obtained upon strain correction approach data acquired in the sweet spot. Differences in diastole are found to be smaller with and without strain correction.

**Figure 5:**
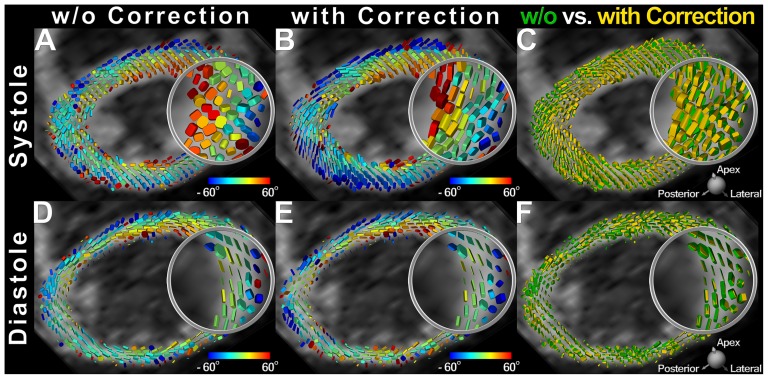
Systolic and diastolic tensor maps with and without strain correction. Diffusion tensor fields acquired in systole (A,B) and diastole (C,D) are represented by superquadric glyphs and color-coded by the helix angle before and (A,C) after strain correction (B,D). The diffusion tensor fields before and after strain correction are merged (C,E) to visualize its impact. Insets demonstrate a major realignment of the tensor field into the typical helical pattern upon strain correction in systole (B). In diastole, strain correction effects are characterized mainly by small changes in the principal diffusivities (E).

In Figure 5 systolic and diastolic tensor fields at a medial level with and without strain correction are compared. The superquadric representation of the diffusion tensor [15] was employed while glyphs were color-coded by the helix angle. It is observed that systolic diffusion tensors have been rearranged into the natural helical alignment after strain correction. In diastole, however, correction effects were subtler, mainly illustrated by small changes in the main diffusivities.

MD and FA for both heart phases, with and without strain correction, are reported in Table 1. After strain correction, the MD was increased in systole and decreased in diastole, both with statistical significance. The FA was significantly increased upon strain correction in systole, but remained unchanged in diastole.

**Table 1:** MD, FA at basal, medial and apical level.

		*systole*		*diastole*	
		*w/o correction*	*with correction*	*w/o correction*	*with correction*
***MD [10^−4^ mm^2^/s]***	*base*	*8.6±1.2* *	*9.5±1.3*	*8.5±1.0* *	*8.2±1.0* †
	*medial*	*8.8±1.4* *	*10.1±1.8*	*9.2±1.1* *	*8.7±1.1* †
	*apex*	*9.6±0.8* *	*11.2±1.2*	*10.1±1.1* *	*9.4±0.9* †
***FA***	*base*	*0.52±0.03* *	*0.61±0.02*	*0.61±0.05*	*0.61±0.04*
	*medial*	*0.52±0.05* *	*0.60±0.03*	*0.61±0.04*	*0.61±0.04*
	*apex*	*0.48±0.02* *	*0.55±±0.02*	*0.57±0.03*	*0.57±0.03* †

* indicates statistical significance (p-value<0.05) between uncorrected and corrected data and

† indicates statistical significance between systole and diastole.

**Figure 6:**
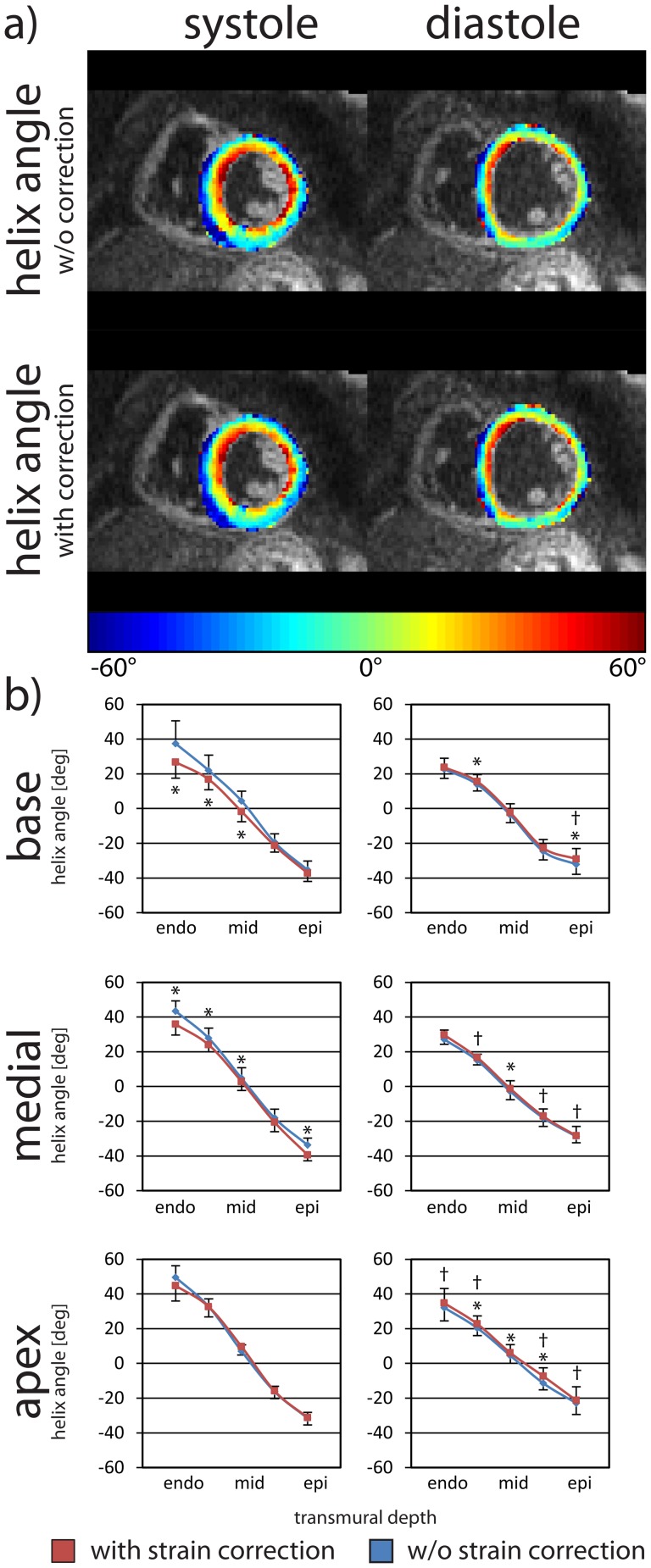
Systolic and diastolic helix angles with and without strain correction. Helix angle maps in systole (left column) and diastole (right column) without and with strain correction (a). The transmural course of the helix angle is given at the basal, medial and apical levels (b). The error bars indicate one standard deviation across the study population. Statistically significant difference between the uncorrected (blue) and the corrected case (red) are indicated by * and between systole and diastole by †.

Figure 6 displays helix angle maps and the dependency of helix angle on the transmural depth for systole and diastole, with and without strain correction. Data are given as mean ± one standard deviation across the study population. While in diastole only little change in helix angles is observed upon strain correction, helix angles at basal level are significantly different with strain correction in systole. The mean transmural helix angle range in diastole across the volunteers was reduced by 2.2±4.4° at the basal level and increased by 2.2±6.1° at the medial level and 1.0±5.0° at the apical level after strain correction. For systole, the transmural helix angle range was decreased by 9.4±9.9° at the basal level, 1.6±5.3° at the medial level and 6.7±9.9° at the apical level after strain correction. Differences in diastole were mostly not statistically significant. In systole, statistically significant differences at the medial and basal levels were found when comparing data without and with strain correction.

**Figure 7:**
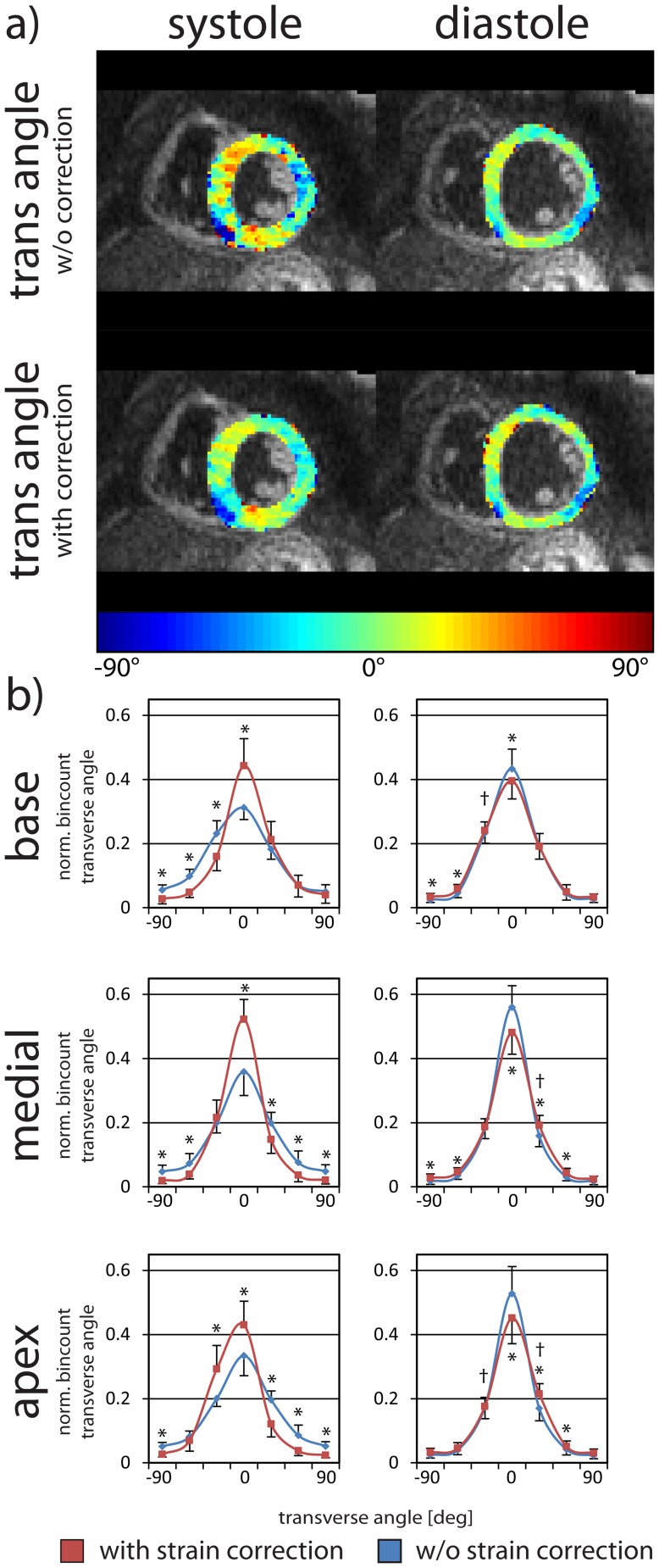
Systolic and diastolic transverse angles with and without strain correction. Transverse angle maps in systole (left column) and diastole (right column) without and with strain correction (a). Transverse angle histograms are given at the basal, medial and apical levels (b). The error bars indicate one standard deviation across the study population. Statistically significant differences between the uncorrected (blue) and the corrected case (red) are indicated by * and between systole and diastole by †.

Figure **7** shows transverse angle maps and histograms of the study population. Significant differences were observed before and after strain correction for both systole and diastole, at each cardiac levels. Negative transverse angles were found at the posterior RV-LV intersection. The distribution of the systolic transverse angle has a lower variance after strain correction suggesting a more coherent fiber track. It is noted that transverse angle distributions in systole and diastole show a similar variance after strain correction.

**Figure 8:**
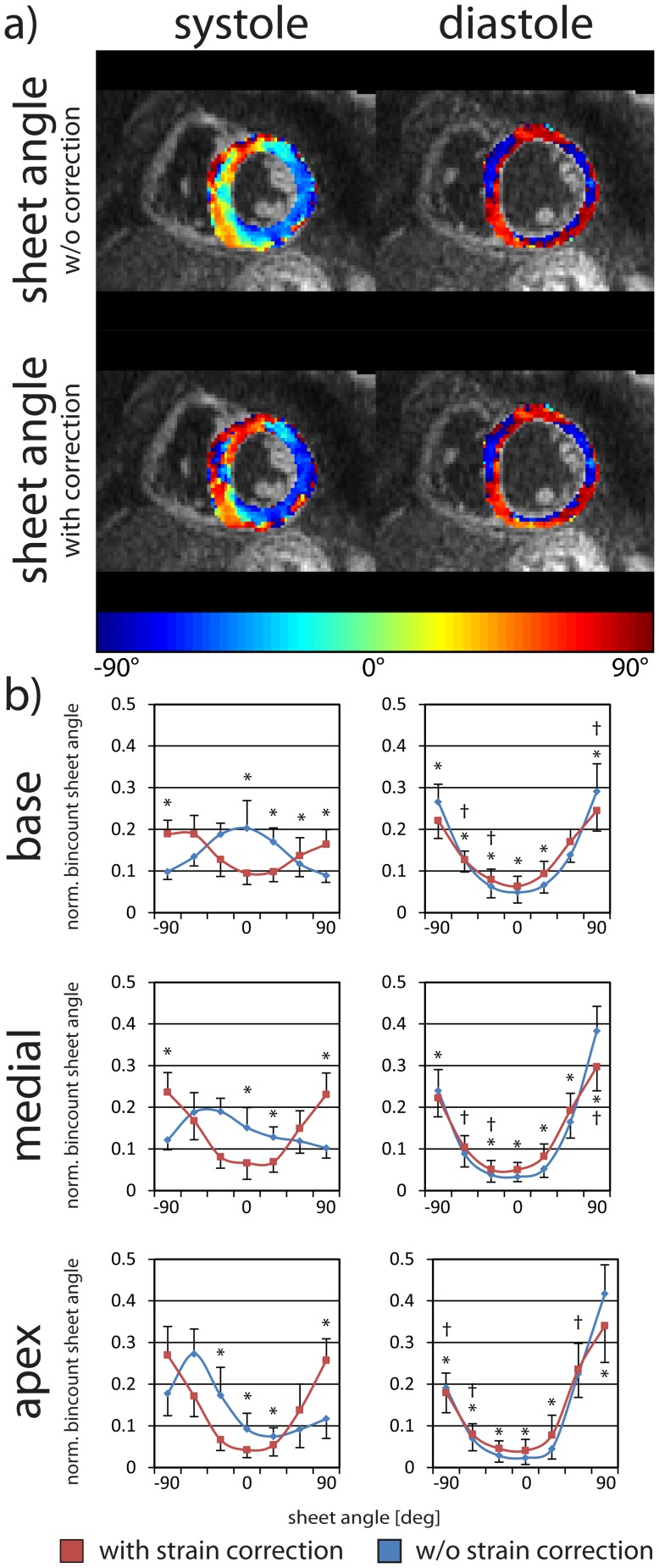
Systolic and diastolic sheet angles with and without strain correction. Sheet angle maps in systole (left column) and diastole (right column) without and with strain correction (a). Sheet angle histograms are given at the basal, medial and apical levels (b). The error bars indicate one standard deviation across the study population. Statistically significant differences between the uncorrected (blue) and the corrected cases (red) are indicated by * and between systole and diastole by †.

Sheet angle maps and histograms are shown in Figure 8. The characteristic distribution of sheet angle into two populations is well seen, particularly with strain correction. Strain correction is observed to change systolic sheet distributions markedly, in some cases producing almost inverted distributions of those obtained without correction.

A comparison of helix, transverse and sheet angles for the systolic and diastolic heart phase with and without strain-correction is provided in Table 2. Significant differences in helix angle range between systole and diastole were seen in the medial and apical levels while differences for transverse and sheet angles between systole and diastole were found at the medial and basal levels of the heart. Sheet angle histograms are broadened in systole compared to diastole.

**Table 2:** Angulation analysis.

			*systole*		*diastole*	
			*w/o correction*	*with correction*	*w/o correction*	*with correction*
***helix angle***	***range***	*base*	*72.8°±15.4°* *	*63.9°±11.5°*	*55.3°±9.3°*	*53.0°±9.6°*
		*medial*	*77.1°±7.7°*	*75.2°±6.2°*	*55.9°±3.7°*	*58.1°±6.1°* †
		*apex*	*81.1°±12.3°*	*76.0°±10.6°*	*55.0°±11.9°*	*56.0°±13.0°* †
	***gradient [*** *°* ***/%depth]***	*base*	*−0.93±0.18* *	*−0.83±0.13*	*−0.74±0.11*	*−0.72±0.11*
		*medial*	*−1.00±0.11*	*−0.98±0.10*	*−0.73±0.06*	*−0.75±0.07* †
		*apex*	*−1.06±0.15*	*−1.00±0.12*	*−0.71±0.14*	*−0.71±0.16* †
***transverse angle***	***mean***	*base*	*−3.0°±4.5°* *	*3.5°±4.8°*	*−0.9°±2.3°*	*−1.8°±2.3°* †
		*medial*	*0.2°±4.3°*	*−1.7°±3.1°*	*−0.4°±1.1°*	*−0.4°±1.1°*
		*apex*	*0.2°±3.05°* *	*−6.3±5.1°*	*1.3°±2.3°*	*1.3°±2.3°* †
	***SD***	*base*	*36.0°±3.8°* *	*29.5°±5.1°*	*27.7°±3.6°* *	*30.2°±3.4°*
		*medial*	*34.0°±5.5°* *	*24.6°±4.1°*	*24.4°±3.1°* *	*27.2°±3.5°*
		*apex*	*35.8°±3.4°* *	*27.3°±2.4°*	*25.2°±4.3°* *	*28.9°±3.8°*
***sheet angle***	***quadratic fit [×10^−^6^^***]******	*base*	*−5.33±3.9* *	*3.7±4.2*	*14.9±4.9* *	*10.6±3.9* †
		*medial*	*−1.44±4.7* *	*9.3±4.5*	*18.8±3.7* *	*14.0±4.3*
		*apex*	*3.5±5.5* *	*12.6±5.7*	*19.2±4.8* *	*14.2±6.7*
	***mean |γ|***	*base*	*38.0°±4.3°* *	*50.4°±3.5°*	*61.2°±5.1°* *	*56.9°±4.2°* †
		*medial*	*41.6°±3.7°* *	*55.1°±4.8°*	*65.0°±3.6°* *	*60.4°±4.3°* †
		*apex*	*58.7°±5.9°* *	*60.5°±4.1°*	*66.0°±4.6°* *	*60.9°±6.6°*

Helix transverse and sheet angle analysis at basal, medial and apical level is shown prior and after strain correction.

* indicates statistical significance (p-value <0.05) between uncorrected and corrected data and

† indicates statistical significance between systole and diastole.

## Discussion

In this study, dual-heart phase cardiac DTI with strain correction was successfully implemented and applied on 10 healthy volunteers to study differences in myofiber architecture between systole and diastole.

The slice and phase interleaving scheme permitted a reduction in scan time by a factor of two relative to a single-phase DTI protocol, which would need to be repeated in systole and diastole. Given that angular diffusion resolution was encoded in separate breath held scans with the current implementation, the number of breath holds required per dual-slice set is dictated by the number of diffusion directions. While breath hold durations were short (14–16 sec), free-breathing acquisition is nevertheless preferred to increase acceptance in practice. To this end, respiratory navigation in conjunction with patient feedback could be incorporated into our approach [27], or alternatively a modified respiratory navigation scheme to increase gating efficiency without the need for patient feedback [40] may be applied.

Local-look excitation was used to reduce the field-of-view in phase-encode direction by a factor of 2.5 to 3 depending on slice angulation and patient size. Alternatively, undersampling strategies may be employed including parallel imaging [55]–[57] or compressed sensing [58], [59]. Further reduction of scan time could also be achieved by combining the proposed method with simultaneous excitation of multiple slices and subsequent unfolding using parallel imaging principles [60]–[62].

DTI of the heart with the stimulated echo approach has previously been performed at sweet spots in the cardiac cycle, where the effects of strain are eliminated [21], [22]. The exact locations of these sweet spots is a function of the heart rate of each volunteer, but generally falls within mid-systole and mid-diastole. More recently, DTI of the myocardium has been described at end-systole, where the heart reaches a quiescent or stand-still phase [27], [41], [44]. With the present work, it has, however, demonstrated that DTI of the myocardium at end-systole is significantly influenced by strain. The effect of strain on helix angle measurements is small but its impact on measures of sheet architecture, such as sheet angle, is extremely large. Our results confirm those of Tseng and colleagues, who likewise showed that imaging away from the systolic/diastolic sweet spots produced small differences in helix angle but very large differences in sheet angle [21], [22].

The validity of strain correction was verified by comparing data acquired in systole and diastole to data obtained in the systolic sweet spot, for which actual material strain equals the average strain across the cardiac cycle. While no major difference was found between the diastolic data obtained without strain correction, and the sweet spot data, significant change was seen for systolic data upon strain correction.

In systole, longitudinal and circumferential diffusion components are underestimated while radial diffusion components are overestimated without strain correction. This effect leads to stretching of the diffusion tensor in radial direction and compression in the two orthogonal directions. Consequently, after estimating the tensors' eigenbasis, the second and third eigenvectors are swapped leading to higher bin counts for the sheet angle around 0°. The first eigenvector, which is predominantly aligned within the circumferential and longitudinal plane, is rotated out of plane. Histograms of the transverse angle demonstrate a wider spread prior to strain correction. Without strain correction, the change in sheet angulation between systole and diastole is significantly overestimated and fiber tracks appear less coherent. Here, the need for strain correction in systole was clearly demonstrated.

The changes in fiber configuration between systole and diastole seen in this study indicate a greater longitudinal alignment of myofibers during contraction. Similar results have been described in excised rat hearts arrested in systole and diastole [33]. Likewise, histological and MR findings from excised porcine [63] and goat [12] heart revealed that the helical fiber structure from epicardium at the apex crosses to endocardium at a medial level and back to epicedium at the base, hypothesizing that the presence of non-zero transverse angles are responsible for wall thickening during contraction [63]. These findings are in agreement with the data reported here.

In the present work sheet angle histograms were generated for basal, medial and apical levels similar to work by Hales et al. [34]. The *in vivo* results presented here show a significant change in sheet angle from systole to diastole. In the contracted state, fewer counts of larger angulation were found, while the counts of intermediate angles were increased. These results are in accordance with those of Dou et al. al [22], who showed that sheet orientation becomes more radial in systole. Similar to prior reports on isolated hearts [33]–[35], the changes in sheet angle histograms were most pronounced at the basal level and less at the apical level.

A potential study limitation lies in the intrinsic coupling of the b-value of the STEAM approach with the subject's heart rate. While the standard deviation of heart rate between acquisitions was only 2.6±1.1 bpm, the heart rate during a breath hold maneuver may have changed significantly. To minimize the impact of heart rate variation on the tensor directionality, all averages of a single diffusion encoding direction were acquired within a single breath hold.

Besides material strain, base SNR is of critical importance regarding systematic errors in determining apparent diffusion. Magnitude averaging of the low SNR DWI data was performed resulting in a Rician noise distribution of the averaged data. Accordingly, signal attenuation by diffusion is biased by the SNR dependent noise floor [64]. Since the SNR at the apex is considerably higher as compared to the base of the heart due to its proximity to the receive coil array, the relative underestimation of apparent diffusion measured in the apex is less compared to the value at the basal level.

## Conclusion

An approach for dual-phase cardiac DTI with correction for myocardial strain has been successfully implemented and has allowed changes in myofiber architecture between systole and diastole to be studied in the human heart *in vivo*. The results obtained with strain correction are in agreement with experimental *ex vivo* data and prior *in vivo* data in healthy volunteers. The potential of DTI to characterize myocardial anatomy in the heart is high, but strain correction at phases other than the sweet spots will be crucial for the accurate characterization of myocyte architecture.
